# The dual pro-inflammatory and bone-protective role of calcitonin gene-related peptide alpha in age-related osteoarthritis

**DOI:** 10.1186/s13075-023-03215-3

**Published:** 2023-12-15

**Authors:** Alexander Hildebrandt, Tamara Dietrich, Jérôme Weber, Mara Meyer Günderoth, Sijia Zhou, Florian N. Fleckenstein, Shan Jiang, Tobias Winkler, Georg N. Duda, Serafeim Tsitsilonis, Johannes Keller, Tazio Maleitzke

**Affiliations:** 1 Charité – Universitätsmedizin Berlin, corporate member of Freie Universität Berlin and Humboldt-Universität Zu Berlin, Center for Musculoskeletal Surgery, Berlin, Germany; 2 Berlin Institute of Health at Charité – Universitätsmedizin Berlin, Julius Wolff Institute, Berlin, Germany; 3 Department of Diagnostic and Interventional Radiology, Charité – Universitätsmedizin Berlin, corporate member of Freie Universität Berlin and Humboldt-Universität Zu Berlin, Berlin, Germany; 4 Berlin Institute of Health at Charité – Universitätsmedizin Berlin, BIH Biomedical Innovation Academy, BIH Charité Clinician Scientist Program, Berlin, Germany; 5 Department of Trauma and Orthopedic Surgery, University Medical Center Hamburg-Eppendorf, Hamburg, Germany; 6 Berlin Institute of Health Center for Regenerative Therapies, Berlin Institute of Health at Charité – Universitätsmedizin Berlin, Berlin, Germany; 7 Department of Orthopaedic Surgery, Copenhagen University Hospital - Amager and Hvidovre, Hvidovre, Denmark; 8 Department of Clinical Medicine, University of Copenhagen, Copenhagen, Denmark

**Keywords:** Calca, CGRP, Pain, Joint inflammation, Cartilage, Bone, Nociception

## Abstract

**Background:**

The vasoactive neuropeptide calcitonin gene-related peptide alpha (αCGRP) enhances nociception in primary knee osteoarthritis (OA) and has been shown to disrupt cartilage and joint integrity in experimental rheumatoid arthritis (RA). Little is known about how αCGRP may alter articular structures in primary OA. We investigated whether αCGRP modulates local inflammation and concomitant cartilage and bone changes in a murine model of age-dependent OA.

**Methods:**

Sixteen- to 18-month-old αCGRP-deficient mice (αCGRP^−/−^_aged_) were compared to, first, age-matched wild type (WT_aged_) and, second, young 4- to 5-month-old non-OA αCGRP-deficient (αCGRP^−/−^_CTRL_) and non-OA WT animals (WT_CTRL_). αCGRP levels were measured in serum. Knee and hip joint inflammation, cartilage degradation, and bone alterations were assessed by histology (OARSI histopathological grading score), gene expression analysis, and µ-computed tomography.

**Results:**

WT_aged_ mice exhibited elevated αCGRP serum levels compared to young WT_CTRL_ animals. Marked signs of OA-induced cartilage destruction were seen in WT_aged_ animals, while αCGRP^−/−^_aged_ mice were mostly protected from this effect. Age-dependent OA was accompanied by an increased gene expression of pro-inflammatory *Tnfa*, *Il1b*, and *Il6* and catabolic *Mmp13*, *Adamts5*, *Ctsk*, *Tnfs11* (*Rankl*), and *Cxcl12*/*Cxcr4* in WT_aged_ but not in αCGRP^−/−^_aged_ mice. αCGRP-deficiency however further aggravated subchondral bone sclerosis of the medial tibial plateau and accelerated bone loss in the epi- and metaphyseal trabecular tibial bone in age-dependent OA.

**Conclusions:**

Similar to its function in experimental RA, αCGRP exerts a dual pro-inflammatory and bone-protective function in murine primary OA. Although anti-CGRP treatment was previously not successful in reducing pain in OA clinically, these data underline a crucial pathophysiological role of αCGRP in age-related OA.

**Supplementary Information:**

The online version contains supplementary material available at 10.1186/s13075-023-03215-3.

## Introduction

With a global prevalence of more than 15% in the adult population [1], osteoarthritis (OA) is the third most rapidly growing disease associated with disability [2], affecting more than 500 million people globally [3]. Intraarticular micro- and macro-injuries trigger reparation processes that initiate pro-inflammatory immune cascades which contribute to progressive and irreversible joint destruction. The vicious cycle of inflammation and tissue damage results in cartilage degradation, pathological subchondral bone remodeling, and synovitis [4], causing debilitating pain, loss of mobility, and decreased quality of life.

Calcitonin gene-related peptide (CGRP) is a nociceptive neuropeptide that contributes to pain perception and sensitization in OA [5]. The 37 amino acid peptide is a member of the calcitonin (CT) peptide family and exists in two isoforms, αCGRP and βCGRP. Whereas αCGRP is encoded by the gene *Calca* and, among other tissues, expressed in the central and peripheral nervous system, βCGRP is encoded by the gene *Calcb* and primarily expressed in the intestine [6]. CGRP can be found in intraarticular perivascular sensory nerve fibers [5] and synovial fluid [7] of osteoarthritic joints. It has both, nociceptive/sensory and efferent/effector functions and arthritic pain develops partially through CGRP-mediated neurogenic vasodilation and inflammation [8]. The density of intraarticular CGRP-positive perivascular nerve fibers is positively correlated with OA severity [5].

We previously showed that αCGRP exhibits an independent pro-inflammatory role in antibody-mediated experimental rheumatoid arthritis (RA) [9]. While RA is however a systemic inflammatory auto-immune disease, OA is characterized by local low-grade inflammation with moderately elevated pro-inflammatory proteins in the plasma and synovial fluid [10]. Interestingly, the density of CGRP-positive nerve fibers is reportedly higher in the synovial tissue of knee joints from OA compared to RA patients [11]. Further, intraarticular CGRP can be secreted by fibroblast-like synoviocytes and its expression correlates with pain in OA [12]. Interestingly, CGRP seems to affect cartilage differently depending on the pre-existing phenotype of chondrocytes. In this regard, a chondro-protective and anti-apoptotic response was observed when CGRP was added to healthy chondrocytes, yet when added to OA-derived chondrocytes, collagen formation markers, and glycosaminoglycans were markedly reduced [13]. However, data from a placebo- and celecoxib-controlled clinical trial failed to show relevant pain relief following monoclonal CGRP-antibody therapy in knee OA patients [14]. A pathophysiological role of αCGRP in OA is therefore possible, yet in vivo evidence is scarce [15].

To explore the effects of αCGRP on intraarticular knee and hip joints during primary OA, αCGRP-deficient (αCGRP^−/−^) and wild-type (WT) mice were exposed to age-dependent OA and compared to young control (CTRL) animals. Our results suggest an independent dual role of αCGRP contributing to pro-inflammatory and catabolic changes intraarticularly, while protecting bone structures from sclerosis and erosion.

## Materials and methods

### Animals and naturally occurring (primary) OA

Female αCGRP^−/−^ and WT mice were used for all experiments [16, 17] and backcrossed at least seven times to ensure a pure C57BL/6 J genetic background. Based on age, WT and αCGRP^−/−^ mice were separated in four groups: Young, 4- to 5-month-old WT_CTRL_ (*n* = 10) and αCGRP^−/−^_CTRL_ animals (*n* = 10) and aged, 16- to 18-month-old WT_aged_ (*n* = 10) and αCGRP^−/−^_aged_ mice (*n* = 10). The employed primary OA model is age-dependent and was previously described in animals with a C57BL/6 J genetic background [18, 19]. Aged WT animals develop spontaneous OA-like joint lesions with a prevalence of up to 90% [20–23]. αCGRP^−/−^ mice exhibit a normal skeletal phenotype, but develop mild spontaneous osteopenia, starting at the age of 4–6 months [24]. All animals were kept at a 12 h light/12 h dark cycle, fed a standard diet, and had access to water ad libitum. Ethical approval was obtained by the competent authority.

The body weight was recorded for all animals before euthanasia using a scale (EMB Scale Ø 150 mm, KERN&SOHN GmbH, Germany).

### *α*CGRP serum protein analysis

Thirty microliters of snap frozen serum were analyzed with an ELISA kit (CSB-EQ027706MO, CUSABIO, Houston, TX, USA), according to the manufacturer’s instructions.

### Sample preparation

Both knee and hip joints were isolated when WT_CTRL_ and αCGRP^−/−^_CTRL_ had reached 4 to 5 months and WT_aged_ and αCGRP^−/−^_aged_ 16 to 18 months. Right knee and hip joints were fixed in paraformaldehyde (PFA) 4% for 48 h, washed and stored in phosphate-buffered saline (PBS) for µ-computed tomography (µCT) analysis. Following µCT scanning, samples were decalcified in 25% EDTA for 20 days, dehydrated (TP 1020 Tissue Processor, Leica Biosystems, Germany, protocol: ethanol 70% 1 h, 80% 3 h, 96% 4 h, 100% 7 h, xylol 2,5 h, paraffin 4 h), and embedded in paraffin (Surgipath Paraplast Plus, Leica Biosystems, Germany). Left knee joints were stripped of all muscle and soft tissue and snap-frozen in liquid nitrogen for RNA isolation and gene expression analysis.

### Histology

Two-micrometer coronal sections of knee and hip joints were cut using a microtome (Rotary 3000 Compact, pfm medical, Germany) and stained with methylene blue (MB) and safranin O (SO). The previously established OARSI histopathological scoring system [25] from 0 to 6 was applied (Supplementary Data S1) by two blinded investigators (AH and TM). For the knee joint, each of the four quadrants of the knee joint: medial femoral condyle (MFC), medial tibial plateau (MTP), lateral femoral condyle (LFC), and lateral tibial plateau (LTP) were assessed separately and scored. For the hip joint, the femoral head (FH) and the acetabulum (AC) were assessed separately and scored. A total joint score was calculated as a mean of all individual scores obtained from MB- and SO-stained slides [25].

### qRT-PCR

Snap-frozen knee joints were trimmed to thin corresponding articular surfaces consisting of synovium, cartilage, and the subchondral bone layer. RNA isolation and reverse transcription to complementary DNA (cDNA) were conducted as previously reported [26]. Snap-frozen joint samples were treated as previously described [9] and quantitative real-time polymerase chain reaction (qRT-PCR) was performed on a 384 well-plate reader in a 7900HT Fast Real-Time PCR System (Thermo Fisher). Raw data were analyzed with SDS v2.4 software (Applied Biosystems). Primers were designed as previously described [9] and provided by Eurofins Genomics GmbH. Primer sequences of assessed genes can be found in Supplementary Data S2. Data points for WT_aged_ and αCGRP^−/−^_aged_ mice are displayed as mean fold changes of two pipetted runs for each sample relative to respective CTRL samples which were set to 1 according to the ddct method [27].

### µCT

Knee and hip joints were analyzed and reconstructed *post mortem* by µCT (Skyscan 1172, Bruker, MA, USA,). Parameters were set as follows: 70 kV, 142 µA, slice thickness 5.1 µm, filter 0.5 Al, rotation step 0.2, averaging frames 3, random movement 10. Raw data were reconstructed as previously described [9]. Systemic bone changes were evaluated in proximal tibiae where a volume of interest (VOI) of 1 mm in length was placed around the outer cortical bone layer, starting 0.5 mm below the most distal point of the growth plate. Assessed global bone parameters included bone volume/total volume (BV/TV) in % and bone density in mg hydroxyapatite (HA)/ccm, as well as trabecular bone parameters, including bone surface in µm^2^, trabecular number (Tb.N) in 1/µm, trabecular separation (Tb.Sp) in µm, and trabecular thickness (Tb.Th) in µm. For evaluation of subchondral bone sclerosis, parameters included cortical volume/total volume (Ct.V/TV) in %, subchondral bone, and pore (Bo&Po) density in mg HA/ccm, and average pore diameter (AvgPo.Dm) in µm [28]. For subchondral bone analysis of the hip joint, a VOI was placed around the femoral head, framing a plate of subchondral bone with a thickness of 80 µm. For radiological evaluation of the subchondral knee joint, the MTP and the epiphyseal trabecular bone were analyzed separately. A VOI was placed around the subchondral MTP, using the same technique as for the femoral head (Supplementary Data S3). The tibial epiphysis was evaluated using a separate VOI, excluding the subchondral bone plate, and avoiding the medial and lateral cortical bone and growth plates (Supplementary Data S3). A threshold of 70 mg HA/ccm for trabecular parameters and 80 mg HA/ccm for subchondral bone parameters was set. Bone density is displayed as 3D images using color maps with a maximum value of 130 mg HA/ccm.

### Statistical analysis

We estimated mean OARSI histopathological grading scores of 2 SD ± of 1.0 for WT_aged_, and mean scores of 0.75 for WT_CTRL_ animals. To obtain a power of 0.8 with an α of 0.05 we calculated that 10 animals per group would be necessary to show an effect size of 1.25. Endpoint comparisons between groups were performed in Prism 9 using the Wilcoxon-Mann–Whitney test*.* For group comparisons of the ordinal OARSI histopathological grading score and body weight, a non-parametric Kruskal–Wallis test with Dunn’s test for multiple comparisons was performed. Outliers were included in the analysis. Unless stated otherwise, data are presented as median ± minimum and maximum. Significance was accepted where *p* < 0.05. For data reporting and storage, we followed the internationally established ARRIVE guidelines [29].

## Results

### Serum αCGRP is elevated in OA and αCGRP-deficiency protects knee joints from histological signs of cartilage degradation in age-related OA

**Fig. 1:**
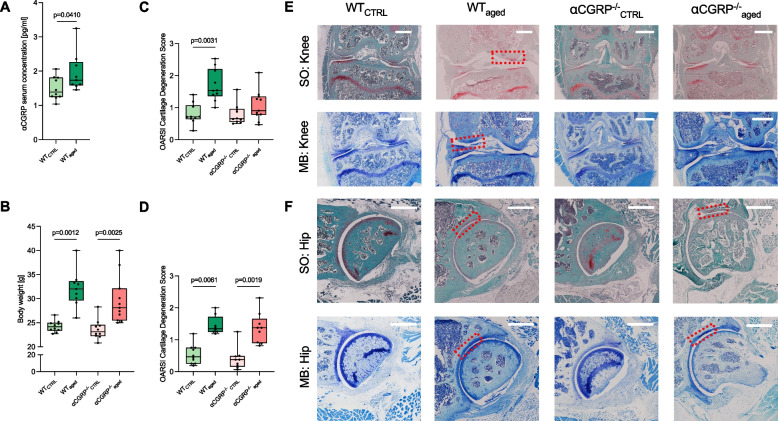
Serum αCGRP is elevated in primary OA and knee but not hip joints of aged αCGRP-deficient mice are protected from cartilage degradation. **A** αCGRP serum concentrations and **B** body weight of indicated groups. **C** OARSI histopathological grading scores of knee and **D** hip joints of indicated groups. **E** Representative histological images of MB- and SO-stained sections of knee and **F** hip joints of indicated groups. Red dotted boxes indicate cartilage damage. Scale bars = 500 µm. Given values are median ± minimum and maximum. MB, methylene blue; SO, safranin O

αCGRP was significantly elevated in serum samples of WT_aged_ compared to WT_CTRL_ animals (*p* = 0.0410) (Fig. 1A).

OA development was accompanied by a significant body weight gain in WT_aged_ (*p* = 0.0012) and αCGRP^−/−^_aged_ animals (*p* = 0.0025) compared to their respective CTRL groups while no significant difference was seen between WT_aged_ and αCGRP^−/−^_aged_ mice (*p* > 0.9999) (Fig. 1B).

In WT_aged_ animals, OA was evidenced by a marked loss of cartilage integrity in knee joints (WT_aged_ vs. WT_CTRL_ *p* = 0.0031), which was also more pronounced when compared to αCGRP^−/−^_aged_ animals, although not statistically significant (*p* = 0.867). Further, no significant difference was seen between αCGRP^−/−^_aged_ and CTRL mice (*p* > 0.9999) (Fig. 1C, E). Overall, knee cartilage loss was most pronounced in the LTP and the MTP (data not shown).

OA was further observed in hip joints of WT_aged_ (*p* = 0.0061) and αCGRP^−/−^_aged_ (*p* = 0.0019) when compared to CTRL animals (Fig. 1D, F). Cartilage loss was evident in both, the FH and the AC (data not shown).

### αCGRP-deficiency prevents overexpression of inflammation markers in OA-affected knee joints

**Fig. 2:**
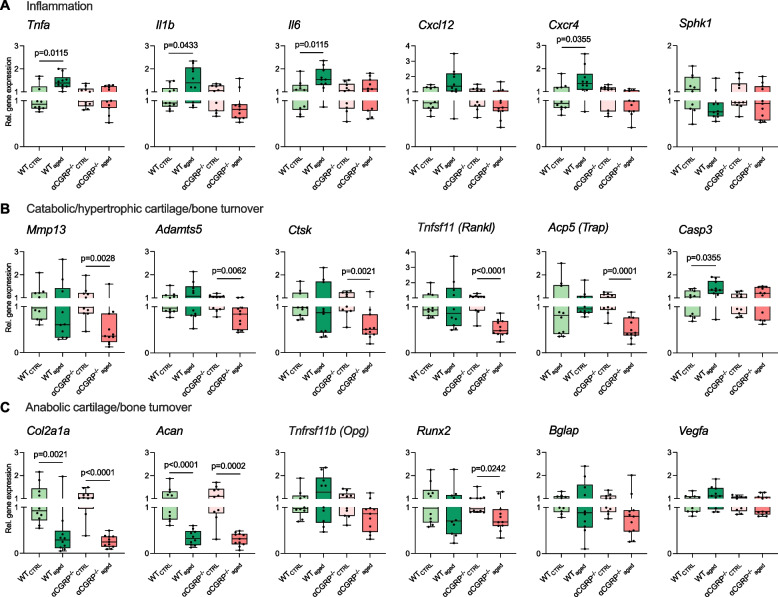
αCGRP promotes intraarticular expression of pro-inflammatory and catabolic cartilage markers in OA-affected knee joints. **A** qRT-PCR gene expression analysis of inflammation markers, **B** catabolic/hypertrophic cartilage/bone turnover markers, and **C** anabolic cartilage/bone turnover markers in knee joint samples of indicated groups. Given values are median ± minimum and maximum. Values for WT_aged_ and αCGRP.^−/−^_aged_ mice are shown as relative fold changes with respect to CTRL groups that were set to 1

To assess molecular gene expression patterns in OA-affected knee joints, qRT-PCR analyses of osseocartilaginous knee joint samples were performed. WT_aged_ mice showed an increased expression of *Tnfa* (*p* = 0.0115), *Il1b* (*p* = 0.0433), and *Il6* (*p* = 0.0115), when compared to WT_CTRL_ mice, while αCGRP-deficient animals were protected from this effect. Pro-inflammatory *Cxcl12* and *Cxcr4* were also increased in WT_aged_ compared to WT_CTRL_ animals, while only reaching statistical significance for the latter (*p* = 0.0355) (Fig. 2A).

### Expression of catabolic cartilage turnover markers is reduced in mice deficient for αCGRP in primary OA

Expression of catabolic cartilage turnover markers *Mmp13* (*p* = 0.0028) and *Adamts5* (*p* = 0.0062), and bone resorption markers *Ctsk* (*p* = 0.0021), *Tnfs11* (*Rankl*) (*p* < 0.0001), and *Acp5* (*Trap*) (*p* = 0.0001) were exclusively reduced in αCGRP^−/−^_aged_ mice compared to CTRLs while *Casp3* was exclusively elevated in WT_aged_ compared to CTRLs (*p* = 0.0355) (Fig. 2B). Further anabolic cartilage turnover markers also decreased with OA development including *Col2a1* (WT_aged_ vs. WT_CTRL_ *p* = 0.0021; αCGRP^−/−^_aged_ vs. αCGRP^−/−^_CTRL_ *p* < 0.0001) and *Acan* (WT_aged_ vs. WT_CTRL_ *p* < 0.0001; αCGRP^−/−^_aged_ vs. αCGRP^−/−^_CTRL_ *p* = 0.0002), however without differences between genotypes. The osteoblast transcription marker *Runx2* was exclusively reduced in αCGRP^−/−^_aged_ mice compared to CTRLs (*p* = 0.0242) (Fig. 2C).

### αCGRP protects from medial tibial subchondral bone sclerosis in age-related OA

**Fig. 3:**
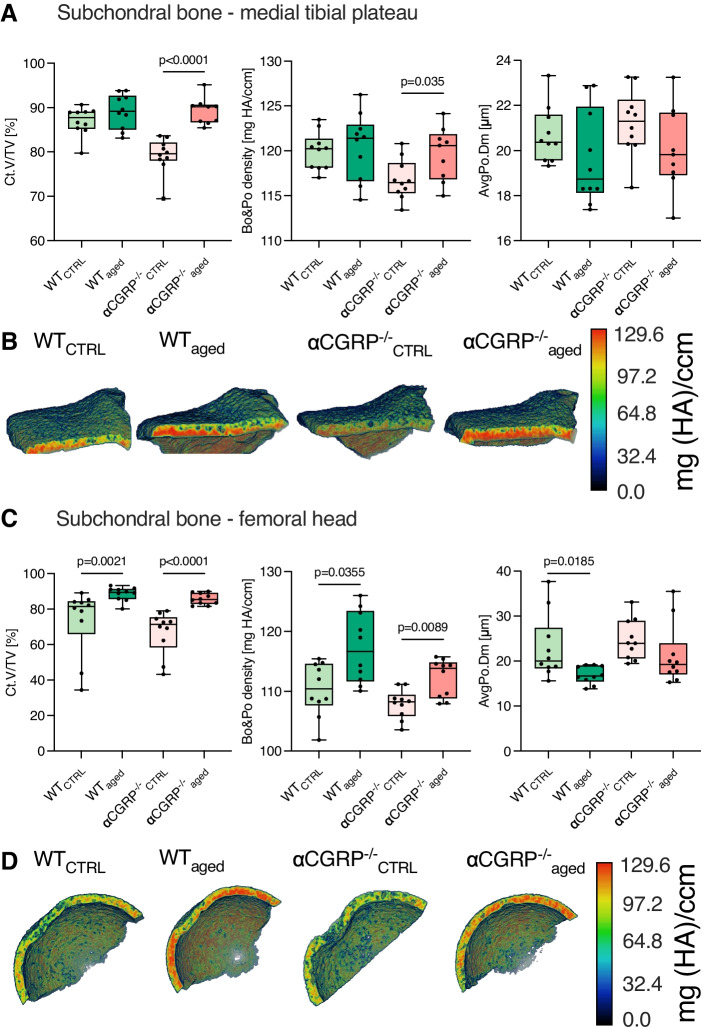
αCGRP prevents the development of primary OA-related subchondral bone sclerosis in the medial knee joint. **A** µCT parameters of the subchondral bone plate of the MTP, and **B** representative 3D images of bone density of the MTP using color maps. **C** µCT parameters of the subchondral bone plate of the femoral head, and **D** representative 3D images of bone density of the femoral head using color maps. Given values are median ± minimum and maximum. Maximum values for color scales were set to 130 mg HA/ccm. MTP, medial tibial plateau

To evaluate OA-induced subchondral bone sclerosis and changes in cortical bone architecture, knee and hip joints were analyzed by µCT. Increased cortical volume (*p* < 0.0001) and subchondral bone density (*p* = 0.035), both indicative of subchondral bone sclerosis of the MTP, were exclusively increased in αCGRP^−/−^_aged_ mice but not in WT_aged_ animals when compared to respective CTRLs (Fig. 3A + B). While cortical volume (WT_aged_ vs. WT_CTRL_ *p* = 0.0021; αCGRP^−/−^_aged_ vs. αCGRP^−/−^_CTRL_ *p* < 0.0001) and subchondral bone density (WT_aged_ vs. WT_CTRL_ *p* = 0.0355; αCGRP^−/−^_aged_ vs. αCGRP^−/−^_CTRL_ *p* = 0.0089) of the femoral head were elevated in both genotypes during OA, subchondral porosity was exclusively decreased in WT_aged_ animals compared to CTRLs (*p* = 0.0185) (Fig. 3C + D).

### αCGRP-deficiency promotes osteopenia and bone sclerosis in the tibial epi- and metaphysis during primary OA

**Fig. 4:**
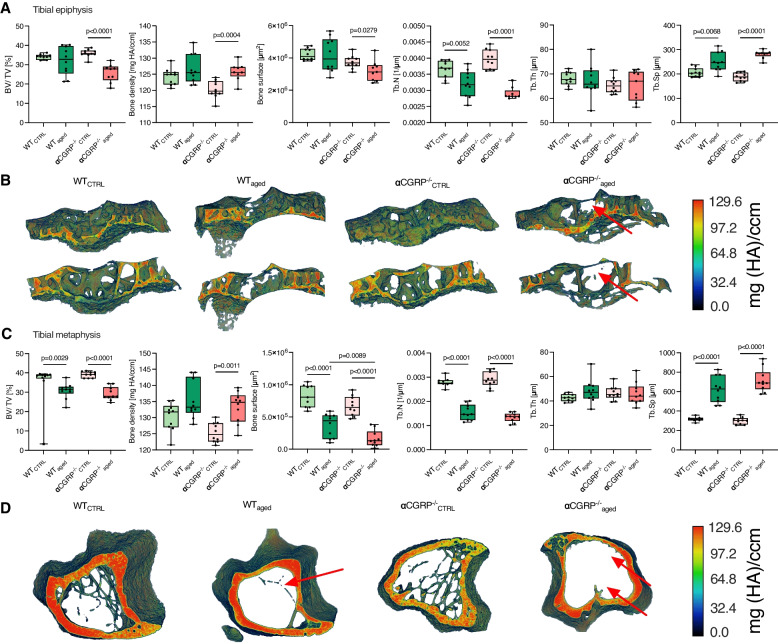
αCGRP prevents radiological OA-related alterations in the tibial epi- and metaphysis. **A** µCT parameters of the tibial epiphysis, and **B** representative 3D images of bone density of the trabecular architecture of the tibial epiphysis using color maps (anterior and posterior sections). **C** µCT parameters of the tibial metaphysis, and **D** representative 3D images of bone density of the trabecular architecture of the tibial metaphysis using color maps. Red arrows indicate loss of trabecular bone mass. Given values are median ± minimum and maximum. Maximum values for color scales were set to 130 mg HA/ccm

To evaluate if age-induced OA further affects osseous structures distal to the subchondral bone, we investigated cortical and trabecular bone structures of the tibial epi- and metaphysis. αCGRP^−/−^_aged_ mice showed marked signs of osteopenia accompanied by sclerotic changes of the tibial epiphysis, while bone integrity was preserved in WT_aged_ mice. In particular, decreased bone volume (*p* < 0.0001), bone surface (*p* = 0.0279), and trabecular number (*p* < 0.0001) and increased bone density (*p* = 0.0004) and trabecular separation (*p* < 0.0001) were observed in αCGRP^−/−^_aged_ animals compared to CTRLs (Fig. 4A + B).

In the tibial metaphysis, bone density was also exclusively increased in αCGRP^−/−^_aged_ mice (*p* = 0.0011), while bone volume was reduced in both genotypes during OA (WT_aged_ vs. WT_CTRL_ *p* = 0.0029; αCGRP^−/−^_aged_ vs. αCGRP^−/−^_CTRL_ *p* < 0.0001) (Fig. 4C + D). Bone surface was lower in αCGRP^−/−^_aged_ than in WT_aged_ mice (*p* = 0.0089), while a loss of bone surface was seen in both genotypes compared to CTRLs (*p* < 0.0001 for both). Trabecular deterioration was present in all aged animals when compared to CTRL mice, indicated by increased trabecular separation and decreased trabecular numbers (*p* < 0.0001 for both genotypes).

## Discussion

In this study, the lifelong absence of αCGRP prevented cartilage degradation of the knee joint and decreased the expression of pro-inflammatory and catabolic cartilage markers in mice suffering from age-induced OA. Yet, aged αCGRP-deficient mice showed distinct signs of tibial subchondral bone sclerosis, impaired bone quality of the epi- and metaphysis, and marked trabecular bone loss. Similar to the observed effects in experimental RA [9], we found αCGRP to display a dual pro-inflammatory and bone protective role in primary, age-dependent OA.

Anti-CGRP therapy has recently been clinically introduced for the prevention and treatment of migraine [30]. Although the pharmacological blockade of CGRP was unable to provide clinically meaningful pain reduction in knee OA patients in a double-blind and placebo-controlled clinical trial [14], an independent pathological role of CGRP in OA—beyond its nociceptive function—is currently being debated [13, 15, 31].

While most in vivo studies employ surgically induced OA models [15, 31] to examine CGRP in OA, this study investigated the role of αCGRP in a murine model of naturally occurring primary OA, which resembles the most common form of OA to date [32].

We were previously able to show that αCGRP-deficiency is associated with decreased intraarticular expression of *Tnfa*, *Il1b*, and *Mmp13* in animals suffering from experimental RA [9]. Here, we again found elevated expression levels of *Tnfa*, *Il1b*, and *Il6* in knee joints of WT_aged_ mice while αCGRP^−/−^_aged_ animals were protected from increased gene expressions of pro-inflammatory cytokines. TNFα, IL1β, and IL6 all contribute to low-grade inflammation and progressive cartilage loss in primary OA [4]. Chondrocytes show reduced proliferation when exposed to IL1β, which further increases the expression of catabolic markers (*Adamts5* and *Mmp13*) and decreases the expression of anabolic markers (*Col2a1* and *Acan*) in cartilage matrix [33]. Elevated serum concentrations of TNFα and IL6 further correlate with radiographic loss of cartilage volume in human knee OA [34].

Binding of the chemokine CXCL12/SDF-1 to its receptor CXCR4 upregulates MMP13, prevents apoptosis of intraarticular leukocytes, and has pro-sclerotic properties in OA-affected joints [35]. SDF-1 is elevated in the synovium of OA patients [36] and pharmacological disruption of SDF-1/CXCR4 signaling leads to a partial attenuation of cartilage damage in preclinical primary OA [37]. In line with these data, we detected an increased expression of SDF-1/CXCR4 signaling in WT_aged_ mice which was not the case in aged αCGRP-deficient mice.

Naturally occurring deterioration of cartilage is observed in mice between 3 and 9 months [20]. Previous in vitro data showed a chondroprotective effect of αCGRP in healthy chondrocytes but a contribution to cartilage deterioration in OA-altered chondrocytes [13]. Accordingly, we saw a protection from histological signs of cartilage destruction and a reduction of catabolic cartilage markers in αCGRP^−/−^_aged_ mice exclusively, while anabolic *Col2a1* and *Acan* were reduced in αCGRP^−/−^_aged_ and WT_aged_ mice alike. In line with our findings, Nakasa et al. showed that blocking CGRP pharmacologically reduced *Mmp13* expression levels and OA progression in vivo [31].

Cathepsin K was previously shown to promote cartilage degradation [38], and mice deficient in cathepsin K were partially protected from surgically induced OA [39]. In addition, receptor activator of NF-κB ligand (RANKL) and Runx2 are overexpressed in OA cartilage [40, 41], and the cell-type specific deletion of Runx2 in chondrocytes protects from experimental OA [42]. Our study showed that αCGRP deficiency led to a marked decrease of *Mmp13*, *Adamts5*, *Ctsk*, *Tnfsf11* (*Rankl*), and *Runx2*, which further underlines the catabolic role of αCGRP for cartilage in primary OA.

While TRAP is traditionally understood an osteoclast marker, an intraarticular role for TRAP in cartilaginous tissues is being discussed. TRAP-positive chondroclasts were previously identified as cells capable of resorbing mineralized cartilage [43] and serum-TRAP was proposed as a clinically relevant and pain-associated biomarker for OA monitoring [44]. We observed a marked reduction of *Acp5* (*Trap*) in aged OA mice deficient for αCGRP, suggesting a protection from catabolic and pro-inflammatory cartilage changes through an inactivation of αCGRP.

As cartilage damage worsens during the course of OA, subchondral bone remodeling is initiated, causing increased sclerotic bone formation and ossification [45]. There is compelling evidence that subchondral bone stiffening further increases mechanical stress to the overlying remaining articular cartilage [46]. In this study, αCGRP^−/−^_aged_ mice showed pronounced signs of subchondral bone sclerosis of the MTP while WT_aged_ animals were partially protected from this effect. The limited extent of sclerotic changes of the MTP in WT mice was previously reported for surgically [15] and age-dependent OA [18, 47]. Together, these findings go in accordance with a reported subtle pro-sclerotic tendency in both, αCGRP-deficient mice receiving destabilizing meniscus surgery and animals receiving sham knee surgery [15]. As immunohistological data showed that the subchondral bone plate of the proximal tibia of healthy rat knee joints contains CGRP-positive nerve fibers [48] this may explain the observed bone-protective effect of αCGRP.

While some authors argue that bone sclerosis in knee OA is limited to the subchondral bone plate [45, 49], recent findings suggest that the epiphysis is also affected by OA-induced defects of the subchondral bone plate, causing a subsequent deterioration of the trabecular architecture beneath [50]. We saw an impaired epiphyseal and metaphyseal trabecular bone structure in all OA animals; however, αCGRP-deficient OA mice exclusively exhibited reduced bone volume and surface with an increased bone density of the epi- and metaphysis. As αCGRP^−/−^ mice develop mild spontaneous osteopenia with age [24], bone deterioration of the subchondral metaphyseal-, and potentially epiphyseal bone may be attributed to the genetically altered skeletal phenotype, while the increased sclerosis of the subchondral bone plate is likely to be caused by an interaction of αCGRP and OA.

Treatment with galcanezumab, an antibody against CGRP, was previously not successful in human OA [14]. The results of this well-conducted clinical trial were surprising, as a previously published preclinical study using the same antibody had shown a significant reduction in pain-related behavior in monoiodoacetate (MIA) -induced and meniscal tear (MT) -induced OA [51]. One potential reason for the observed differences is the choice of preclinical OA model. While the clinical trial was conducted in primary OA patients, all pre-clinical data were based on two secondary OA models [51]. The difference between primary and secondary OA is well researched as recently laid out by Poulsen et al., 2023 [52]. Further, current research in the OA field moves away from understanding OA as one disease and instead strives to identify different endo- and phenotypes which are likely to respond differently to available and newly developed treatments. This may explain in part why numerous “successful” preclinical studies can rarely confirm their findings clinically [53]. Interestingly, galcanezumab has not been tested in a primary OA model before.

We showed that a lifelong blockade of αCGRP signaling alleviates naturally occurring OA in female mice. Our results imply that the inhibition of αCGRP signaling could be a promising therapeutic approach with translational potential in OA therapy when applied to the right disease and at the right disease stage.

The current study has several limitations. First, the radiological data sets do not allow to distinguish between OA-induced subchondral bone changes and calcified cartilage. The diameter and density of both, calcified cartilage and subchondral bone, increase similarly during progression of OA [15, 45]. This additional information has thus little relevance for the data presented in our study. Second, while joint samples were carefully and precisely dissected, the employed gene expression analyses lack tissue specificity, as samples were comprised of a mixture of cartilage, synovium, and small amounts of subchondral bone. When processing murine joint samples, a selective tissue examination is technically difficult, as joint samples are commonly crushed, minced, or digested [9, 12]. Third, we were only able to report changes that occurred until or during end-stage OA. OA is however a multi-stage disease with various origins and courses. This needs to be taken into consideration when interpreting the results. Fourth, our data did not include human samples. A lifelong blockade of αCGRP signaling, as utilized in our experiment, is an artificial model lacking direct translational potential. Further studies must include human samples, longitudinal disease evaluation, and pharmacological inhibition of αCGRP must be tested in primary and secondary OA. And finally, due to the higher prevalence of OA in women [1], we exclusively investigated female mice. The deterioration of estrogen activity during aging may however have impacted bone and cartilage quality additionally [54], warranting further studies on the role of αCGRP in the male organism.

## Conclusions

In this study, we demonstrated that lifelong disruption of endogenous αCGRP-signaling protected animals from histological and molecular signs of cartilage degradation in primary, age-dependent OA. αCGRP serum levels were increased in primary OA and the inactivation of αCGRP impeded OA-associated overexpression of intraarticular inflammation and catabolic cartilage markers including IL1β, IL6, TNFα, MMP13, ADAMTS5, Cathepsin K, RANKL, RUNX2, and SDF-1/CXCR4 in joint tissues. In contrast, lack of αCGRP was associated with subchondral bone sclerosis and tibial osteopenia in primary OA. Although the clinical application of anti-CGRP treatment did previously not meet the primary endpoint of pain reduction in knee OA patients, our study provides first-hand evidence for a dual pro-inflammatory and bone-protective role of αCGRP in naturally occurring OA.

### Supplementary Information

**Additional file 1: S1.** OARSI histopathological grading score. **S2.** Primer sequences. **S3.** Assessment of µCT parameters.

## Data Availability

The datasets used and/or analyzed during the current study are available from the corresponding author on reasonable request.

## Abbreviations

AC: Acetabulum
αCGRP: Calcitonin gene-related peptide alpha
AvgPo.Dm: Average pore diameter
BV/TV: Bone volume/total volume
CT: Calcitonin
cDNA: Complementary DNA
CTRL: Control
Ct.V/TV: Cortical volume/total volume
FH: Femoral head
HA: Hydroxyapatite
LFC: Lateral femoral condyle
LTP: Lateral tibial plateau
MFC: Medial femoral condyle
MTP: Medial tibial plateau
MIA: Monoiodoacetate
MT: Meniscal tear
MB: Methylene blue
OA: Osteoarthritis
PBS: Phosphate-buffered saline
qRT-PCR: Quantitative real-time polymerase chain reaction
RANKL: Receptor activator of NF-κB ligand
RA: Rheumatoid arthritis
SO: Safranin O
Bo&Po: Subchondral bone and pore
Tb.N: Trabecular number
Tb.Sp: Trabecular separation
Tb.Th: Trabecular thickness
VOI: Volume of interest
WT: Wild-type
µCT: µ-Computed tomography
