# Supplementary material for: The dual pro-inflammatory and bone-protective role of calcitonin gene-related peptide alpha in age-related osteoarthritis
Source: Arthritis Res Ther. 2023 Dec 15;25:244. doi: 10.1186/s13075-023-03215-3 (PMC10722726; doi:10.1186/s13075-023-03215-3)
Supplement: Supplementary file 1 — Additional file 1: S1. OARSI histopathological grading score. S2. Primer sequences. S3. Assessment of µCT parameters. [file 13075_2023_3215_MOESM1_ESM.docx]

**Supplementary Data**

**S1 – OARSI histopathological grading score:**

Score 0=Normal; Score=0.5: Loss of Safranin-O without structural changes; Score=1: Small fibrillations without loss of cartilage; Score=2: Vertical clefts down to the layer immediately below the superficial layer and some loss of surface lamina; Score=3: Vertical clefts/erosion to the calcified cartilage extending to <25% of the articular surface; Score=4: Vertical clefts/erosion to the calcified cartilage extending to 25–50% of the articular surface; Score=5: Vertical clefts/erosion to the calcified cartilage extending to 50–75% of the articular surface; Score=6: Vertical clefts/erosion to the calcified cartilage extending >75% of the articular surface.

**S2 – Primer sequences:**

*Acan* forward CAATTACCAGCTGCCCTTCA,

*Acan* reverse CAGGGAGCTGATCTCGTAGC,

*Acp5* forward GGTATGTGCTGGCTGGAAAC,

*Acp5* reverse ATTTTGAAGCGCAAACGGTA,

*Adamts5* forward ACGGCATTATTGGCTCAAAG,

*Adamts5* reverse GGGATCCTCACAACGTCAGT,

*Bglap* forward CCTGGCTGCGCTCTGTCT,

*Bglap* reverse TGCTTGGACATGAAGGCTTTG,

*Casp3* forward GGGAGCAAGTCAGTGGACTC,

*Casp3* reverse CGTACCAGAGCGAGATGACA,

*Col2a1* forward GGTCCCCCTGGCCTTAGT,

*Col2a1* reverse CCTTGCATGACTCCCATCTG,

*Ctsk* forward GTCGTGGAGGCGGCTATATG,

*Ctsk* reverse AGAGTCAATGCCTCCGTTCTG,

*Cxcl12* forward CAGAGCCAACGTCAAGCA,

*Cxcl12* reverse AGGTACTCTTGGATCCAC,

*Cxcr4* forward ACGGCTGTAGAGCGAGTGTT,

*Cxcr4* reverse AGGGTTCCTTGTTGGAGTCA,

*Gapdh* forward ACTGAGCAAGAGAGGCCCTA,

*Gapdh* reverse TATGGGGGTCTGGGATGGAA,

*Il1b* forward ACCTAGCTGTCAACGTGTGG,

*Il1b* reverse TCAAAGCAATGTGCTGGTGC,

*Il6* forward CCCCAATTTCCAATGCTCTCC,

*Il6* reverse CGCACTAGGTTTGCCGAGTA,

*Mmp13* forward GATGGCACTGCTGACATCAT,

*Mmp13* reverse TTGGTCCAGGAGGAAAAGC,

*Rankl* forward GTACGCCAACATTTGCTTTCG,

*Rankl* reverse GTAGGTACGCTTCCCGATGTT,

*Runx2* forward GTGGCCACTTACCACAGAGC,

*Runx2* reverse TGAGGCGATCAGAGAACAAA,

*Sphk1* forward AGGTGGGGCTATGACTTGGA,

*Sphk1* reverse CCCAGGGAAGGTCCCTAAGA,

*Ocn* forward GAGTGTGAGGAAGGGCGTTA,

*Ocn* reverse CAAACTGTGTTTCGCTCTGG,

*Tnfa* forward ACGCTGATTTGGTGACCAGG,

*Tnfa* reverse GACCCGTAGGGCGATTACAG,

*Vegfa* forward TCTCCCAGATCGGTGACAGT, and

*Vegfa* reverse AAGGAATGTGTGGTGGGGAC.

**S3 – Assessment of µCT parameters:**

**S3. Assessment of µCT parameters**. (A) Assessment of BV/TV and bone density in the tibial metaphysis. The VOI includes both the tibial cortex and bone marrow. (B) Assessment of bone surface, Tb.N, Tb.Sp and Tb.Th in the tibial metaphysis. The shrinked VOI spares the tibial cortex. (C) VOI placement for assessment of the MTP and (D) the epiphyseal trabecular bone. The VOI of the tibial epiphysis spares the subchondral plate, lateral and medial cortices, and the growth plate. (E) VOI placement for assessment of the femoral head. Scale bars = 500µm. Yellow lines indicate VOI placements.
